# Knowledge, Attitudes and Behaviours Towards Alcohol Consumption and Cardiovascular Health Among Healthcare Students of South Asian Heritage in the UK: A Qualitative Study

**DOI:** 10.3390/pharmacy13050136

**Published:** 2025-09-18

**Authors:** Jeevan Singh, Samira Osman, Sarah Baig, Yousuf Murad, Zahraa Jalal

**Affiliations:** 1 School of Pharmacy, Institute of Clinical Sciences, College of Medical and Dental Sciences, University of Birmingham, Birmingham B15 2TT, UK; 2 Worcestershire Acute Hospital Trust, Woodrow Dr., Redditch B98 7UB, UK

**Keywords:** South Asians, alcohol, cardiovascular health, pharmacy services

## Abstract

**Background:** Cardiovascular disease is a leading cause of mortality in England, with South Asians estimated to have a higher risk of CVD development compared to the general population. Harmful drinking is a key risk factor for cardiovascular disease, but little is known about drinking behaviours among South Asians, especially those aged 18–25. **Objectives:** The objectives of this study were (i) to investigate the knowledge of the cardiovascular effects of harmful drinking among young South Asians aged 18–25, and (ii) to explore the perceptions of young South Asians towards the role of the pharmacist in supporting individuals with alcohol-related harm. **Methods:** Qualitative, in depth, face-to-face, semi-structured interviews were conducted with ten young South Asians, around 30 min in length. The interviews were audio recorded, transcribed verbatim and then thematically analysed. **Results:** Four superordinate themes emerged: (1) Culture Clash; (2) The Great Escape; (3) Cardiovascular Confusion; and (4) The Ambiguous Pharmacist. These themes highlighted a dichotomy between the drinking behaviours among different South Asian communities, a lack of knowledge regarding the cardiovascular consequences of harmful drinking and mixed views regarding pharmacists’ role in supporting dependence. Participants acknowledged the prevalence of poor cardiovascular health among British South Asians, citing various types of dysfunction and possible causes; however, the effect of drinking specifically was not understood. Most participants would refer a young person struggling with dependence to a pharmacist and would be receptive to discussing drinking with one. However, few commented on any role outside of signposting to other services or healthcare providers. **Conclusions:** Greater cardiovascular health promotion is needed among South Asians, with an emphasis on the link between excess alcohol consumption and cardiovascular dysfunction. Furthermore, pharmacists must do more to promote greater awareness of the different ways in which alcohol dependence can be supported within the community to encourage young people seeking harm reduction to utilise those services as needed.

## 1. Introduction

Cardiovascular disease (CVD) in England alone is responsible for 25% of deaths and estimated to cost the NHS GBP 7.4 billion with an additional economic cost of GBP 15.8 billion [1]. British South Asians are disproportionately affected, with a 29–67% higher risk of CVD, and the possibility of developing coronary heart disease and incident atherosclerotic cardiovascular disease significantly higher than European populations [2,3,4]. Various modifiable and non-modifiable factors contribute to cardiovascular dysfunction; however, harmful alcohol consumption remains a significant risk factor [5]. The UK recorded approximately 280 thousand estimated alcohol-related hospital admissions from 2019 to 2020, showing an 8% increase since 2016, which was associated with over 20 thousand 16–24-year-olds [6]. In 45% of all alcohol-related admissions during this period, cardiovascular diagnoses were found to be the most common primary diagnosis. Diagnoses included arrythmias, cardiomyopathy, hypertension, and heart failure [6]. Anecdotally, alcohol consumption is regarded as ‘embedded’ within British culture with young people in Europe, reporting some of the highest rates of drinking globally [7,8]. However, the literature remains vague about alcohol consumption among South Asians living in the UK and representation within alcohol support services even more so [9]. In this paper, the term ‘South Asians’ refers to individuals with ancestral origins in countries of the Indian subcontinent, including India, Pakistan, Bangladesh, Sri Lanka, Nepal, Bhutan, and the Maldives.

In one of the few multi-ethnic and multi-faith studies investigating alcohol consumption, conducted in the 1990s, Sikh men reported higher average alcohol scale scores compared to their white and Hindu peers and were more likely to be regular drinkers [10,11]. The minority of Muslim men who admitted to drinking reported the highest rate of alcohol consumption overall [10]. Few studies have revisited drinking behaviours among British South Asians; however, those that have been conducted found alcohol-associated hospital admissions were reduced in white men compared to their Indian peers [12]. Within Birmingham, UK, Sikh men are reported to consume more units per week than their White British counterparts [13]. Moreover, a recent demographic report commissioned by Birmingham City Council, reported that Birmingham-based Hindu and Muslim men are increasingly likely to recognise their own harmful drinking than Sikh men. Drinking among second generation Sikh women was also reported to be more popular than it was for earlier generations of women [13].

Second-generation British South Asians are thought to have adopted some healthier behaviours, compared to earlier generations; however, various studies report that second-generation ethnic minorities are increasingly likely to consume more alcohol [14,15]. A recent study of young Sikh men, aged 18–27, found that most of them lacked concern about the social or health consequences of their alcohol consumption and dismissed problematic behaviour if it did not affect others negatively [16]. Dependence was reported to be hidden due to shame and judgement, with many unable to elaborate on where to seek support for harmful drinking. Few were able to list pharmacies as places to access harm reduction [16].

Community pharmacists remain some of the most accessible healthcare providers in Britain; therefore, they have great potential to support young people struggling with alcohol dependence [17]. Typically, adolescents are less likely to recognise dependence and avoid seeking help due to embarrassment, social stigma, lack of awareness or the preference for self-reliance [18]. Current guidance allows pharmacy staff to undertake indicative screening to identify those whose drinking is above low risk [19]. Furthermore, The New Medicine Service facilitates opportunistic discussions between pharmacy staff and patients around new medicines and alcohol use with the provision to provide brief advice and refer as appropriate [19]. Whilst specific guidance tailored to supporting young people struggling with alcohol dependence remains undeveloped, behavioural interventions such as motivational interviewing (MI) and cognitive behavioural therapies (CBT) are the best evidence-based approaches towards harm reduction in young people, especially older adolescents [20,21]. As community pharmacy provisions develop, pharmacists may be required to support harmful drinking in young people through the delivery of MI or CBT. Equally, brief interviewing (BI) is a patient-focussed counselling technique which establishes motives and steps for patients to make towards positive change [22]. Whilst the evidence base for its use in reducing harm among adults is strong, its practice with young people remains novel [23].

In the UK, community pharmacists do not formally treat alcohol dependence but play a growing role in early identification and brief intervention. Pharmacists can conduct indicative screening using validated tools (e.g., AUDIT-C), offer brief advice, and refer individuals to specialist services as needed—activities which align with NHS England’s public health priorities [1]. The New Medicine Service (NMS) is a structured, NHS-commissioned service where pharmacists provide support to patients newly prescribed medicines for long-term conditions. While not designed for alcohol treatment, it creates opportunities for opportunistic alcohol-related conversations, especially when medicines are contraindicated with alcohol [1]. These services are remunerated by the NHS and are widely available in England, yet public awareness remains low, particularly among young adults [24]. Promotion of pharmacy-based alcohol interventions is minimal, and uptake is variable across settings [17]. Greater clarity around service availability and expanded roles; such as pharmacist-led brief interventions or motivational interviewing, could enhance their utility for addressing alcohol-related harm in youth populations.

To date, studies have not investigated the knowledge, attitudes, and behaviours towards harmful drinking and the cardiovascular effects among young British South Asians aged 18–25. Additionally, the perception of pharmacy services to support young people struggling with alcohol dependence in the future, among young South Asians, has not been explored.

This study aims to address these gaps by verifying whether young South Asians living in the UK require increased health promotion, to reduce alcohol-associated cardiovascular conditions in later life. Moreover, this study will determine what young South Asians perceive the role of the pharmacist to be in supporting alcohol dependence in young people in the future and what barriers, if any, would prevent them from accessing harm reduction.

### Aims and Objectives

- To investigate the knowledge of the cardiovascular effects of harmful drinking among young South Asians aged 18–25.
- To explore the perceptions of young South Asians towards the role of the pharmacist in supporting alcohol dependence.

## 2. Materials and Methods

### 2.1. Participants

Eligible participants were recruited using a purposive snowball sampling technique, achieving a small sample of young South Asians (*n* = 10; 7 females, 3 males) [25]. Inclusion criteria: Participants aged 18–25, ethnically South Asian, studying at the University of Birmingham and able to attend a 1:1 face-to-face semi-structured interview, able to converse in English, willing to participate in the study, and able to provide verbal and written consent. All participants were in their second, third or fourth year of undergraduate study. Although recruitment was open to all eligible South Asian students at the University of Birmingham, pharmacy students responded most readily. This represents a limitation in sampling diversity and may have introduced bias in views toward pharmacy services. Participants who did not identify as South Asian or had limited knowledge of the British South Asian diaspora were excluded (see Table 1).

### 2.2. Procedure

Participants were approached directly via email. Upon fulfilling the inclusion criteria, they were asked to notify any potential participants in their social circle. Information sheets were supplied to willing participants and upon receipt of written, informed consent, semi-structured interviews were conducted, around 30 min in length. The interviewing period lasted 4 weeks from mid-October to mid November 2023. Face-to-face interviews were conducted on the university premises. Post-interviewing, participants were thanked for their participation. Literature about the knowledge, attitudes, and behaviours towards the cardiovascular consequences of harmful drinking and perceptions of pharmacy interventions is limited, thus an inductive approach was used. Although participants were recruited through snowball sampling, interviews were conducted individually, and participants were not asked to disclose relationships with other participants. We acknowledge the possibility of shared peer influences as a limitation.

### 2.3. Materials

The semi structured interview guide contained 9 questions, based around 5 areas: (1) knowledge, attitudes and behaviours towards alcohol; (2) the prevalence of drinking among young South Asians; (3) awareness of poor cardiovascular health among South Asians; (4) the relationship between harmful drinking and poor cardiovascular health and the impact of harmful drinking on later life; (5) the perceived role of the pharmacist in supporting young people with alcohol dependence (Appendix A). The interview guide was piloted before use, to ensure clarity and relevance. The responses obtained from the pilot interview have been excluded from this study. Ethical approval was granted by the safety and ethics sub-committee. Ethical approval no. (078R3).

### 2.4. Data Analysis: Thematic Data Analysis of Interview Transcripts

Interviews were audio recorded and transcribed verbatim using Microsoft Word. Transcripts were then thematically analysed, following the principles of Braun and Clarke (2006) [26]. The researcher JS undertook data familiarisation via initial and repeated readings of the transcripts prior to coding and the emergence of themes and subthemes. Upon completion of data familiarisation, all transcripts were analysed by researcher JS, with initial codes developed based on highlighted excerpts. Results were checked by a second researcher ZJ. Related ideas across all transcripts were clustered, producing an initial thematic framework, which was consistently compared, contrasted, and refined to verify the validity of emergent themes (Appendix B).

## 3. Results

Data saturation in qualitative research can be achieved through 9–17 interviews in homogenous populations, ten interviews were conducted with South Asians of various ethnic and religious backgrounds, aged 18–25 [27], and it was determined that saturation was achieved after the tenth interview. Participants included both male and female students, studying either Dentistry, Medicine, or Pharmacy. Participants’ demographic data is presented in Table 1.

### 3.1. Themes

Following thematic analysis, four major themes emerged: 1. Culture Clash; 2. The Great Escape; 3. Cardiovascular Confusion; and 4. The Ambiguous Pharmacist.

#### 3.1.1. Theme 1—Culture Clash

Most participants reported observing members of their family drinking during childhood, as their earliest memory of alcohol, with many consuming their first drink below the age of 18, within the family home or with friends. For example, Participant E stated: *‘I was quite young when I started drinking, maybe just like at family events or something. And I remember I was a lot younger than 18.’* Similarly, Participant J commented: *‘In secondary school, quite a few people from my friendship group would drink quite often and kind of every weekend.’*

Most participants believed that drinking was prevalent among British South Asians but especially popular for British Punjabis:

*‘In the south Asian community, I feel like people drink too much, like in general…I don’t like that part.’*

(Participant B)

*‘So definitely the Punjabi community will drink lots.’*

(Participant H)

Additionally, drinking was regarded as more common among Sikhs and Hindus:

*‘I have some Hindu and Sikh friends and they quite enjoy drinking as a social activity.’*

(Participant J)

However, two participants commented that consumption among many young Muslim men is comparable to other communities in the UK but hidden:

*‘I’ve had quite a few experiences, especially with Pakistani South Asians, specifically Pakistanis. They’ve always been like, don’t tell anybody that I’m drinking…sort of in a jokey way, but you know, it’s not joking. Like, don’t tell anybody.’*

(Participant D)

*‘The younger generation tend to drink just as much if not more than others because they do it less frequently…they probably wouldn’t do it in a family setting.’*

(Participant H)

*‘Pakistanis that I know, their first drink was either borrowing it from a friend’s house or in a field…They drank probably way too much and end up being sick, like violently ill from the amount.’*

(Participant D)

Participants further commented that some women continue to be expected to abstain from alcohol:

*‘If a woman drank alcohol, and it’s still a bit like this, it would be like, oh she’s not wife worthy.’*

(Participant D)

Therefore, a clash exists among different South Asian communities, where consumption is open for some and hidden for others.

#### 3.1.2. Theme 2—The Great Escape

This theme reflected on why young South Asians may engage in harmful drinking. Participants commented on an increased likelihood to engage in risky drinking at university, to rebel against strict upbringings or enjoy a newfound freedom:

*‘I think one of the main reasons is, I feel like for some people, they come from a strict family. They’re like, when they come to university, they’re like, let’s go wild, you know, like party out.’*

(Participant B)

University sports culture and peer pressure from friends and family were also mentioned:

*‘Especially with socials like sports nights and things where you can say, like, people aren’t being forced to drink but you could feel judged if you didn’t.’*

(Participant E)

*‘Peer pressure as well can propagate harmful drinking…specifically within their own families first, if their families may drink and also at university.’*

(Participant F)

Participants reflected the view that young people who observe excess drinking within the home are increasingly likely to follow suit:

*‘I feel like if your parents drink you might adopt it and think ‘Oh, they’re drinking its fine I can drink as well.’’*

(Participant B)

Others, particularly of Punjabi heritage, recalled instances where they feared social exclusion if they refrained from participating, due a cultural expectation to drink:

*‘I feel like in the Punjabi community it’s very strong, like it’s a bit weird if you aren’t drinking, but I wouldn’t say that’s the same for all communities.’*

(Participant C)

Whilst drinking was believed to be for enjoyment, one participant attributed this to the general culture of drinking in Britain:

*‘With second generations, I would say it’s much more of a situation where those people are part of British Culture so it’s probably more for enjoyment.’*

(Participant C)

#### 3.1.3. Theme 3—Cardiovascular Confusion

Most participants believed cardiovascular dysfunction was prevalent, expected and accepted, particularly among older British South Asians. For example, Participant H commented: *‘It’s so widespread and so accepted that the older you get the more issues you have, especially cardiovascular-related.’*

Reasons attributed by participants included: cultural diets, lack of exercise, and inadequate cardiovascular health promotion:

*‘Diet could have a big impact, especially if you consider Indian sweets…they don’t always assume and link it towards their health… its just never crossed their mind…and they don’t always consider things like exercise.’*

(Participant E)

One participant acknowledged seeing some cardiovascular health promotion in the past but was unable to provide any specific examples and most shared the view that awareness among all South Asians remains low:

*‘In terms of awareness, I don’t think there is much, but I’m aware that there are some public health campaigns, but I don’t think that specifically for South Asians, there is very good awareness in my experience.’*

(Participant F)

Participants readily recognised the prospect of poor cardiovascular health due to harmful drinking, and some provided examples of cardiovascular dysfunction. For example, when asked what disease states could be linked to harmful drinking, Participant J commented: *‘heart attacks, clogging up of your arteries or atherosclerosis.’*

However, the direct effect of alcohol on the heart was poorly understood and most were unsure whether they would be able to explain the relationship between the two to a peer:

*‘A two out of ten. Not very confident, because I don’t know parts of cardiovascular health it worsens. I don’t really know the rationale behind it or why it happens so I just wouldn’t feel comfortable sharing.’*

(Participant D)

*‘I’m a four out of 10…I think I lack education, in normal education up to secondary school level where the association between alcohol and cardiovascular health has not really been made clear.’*

(Participant H)

Only two participants commented on lifestyle changes they have implemented to improve their health, for example, Participant G commented:

*‘I used to have four teaspoons of sugar, but I’ve cut that down to two in tea or coffee. So, I do take considerations within my diet and decrease the amount of rice and stuff but I’m not taking active precautions against those specifically. I’m just trying to make sure I’m healthy in general.’*

However, cardiovascular health improvement was not the focus. Similarly, participants reported to regular gym use among young South Asians but argued cardiovascular health is seldom considered:

*‘There’s a whole gym culture right now, but I don’t think that’s like related to protect cardiovascular health it’s more about image and sometimes mental health.’*

(Participant G)

#### 3.1.4. Theme 4—The Ambiguous Pharmacist

Nine participants expressed they would refer a young person displaying harmful drinking to either a GP or a pharmacist, who they deemed to be appropriate due to their skills, training, and education. No consistent gender-based differences were observed in participants’ responses to questions about pharmacy roles or help-seeking behaviour, although the sample size was insufficient to support systematic comparison:

*‘I think they’ve been trained on giving advice to people who are looking to improve on any bad habits they might have, like drinking…and they would probably have a good working knowledge of people suffering from alcohol dependency.’*

(Participant H)

The main benefit suggested of discussing dependence with a pharmacist, however, was accessibility:

*‘I would say go and talk to your GP, or pharmacist because they’re probably the easiest person to talk to as GP appointments are very hard to get.’*

(Participant C)

*‘Look around you, there’s how many community pharmacies and more growing. You don’t need to wake up at 7.45 in the morning and call just to get an appointment to speak to them.’*

(Participant G)

However, participants were mostly unsure what the role of the pharmacist was aside from signposting: ‘their main role would be to signpost to the correct areas where they can go to get deeper, more specific help, because their knowledge might not be that in depth.’ (Participant C).

*‘Maybe some sort of one-to-one session but I don’t know if pharmacists could do that. I’m not sure what the role of the pharmacist could be.’*

(Participant G)

One participant, who objected to referring alcohol dependence to a pharmacist, expressed lack of confidence in the ability of pharmacists to help:

*‘I don’t feel like they really do anything apart from put up random posters.’*

(Participant B)

Several barriers which could prevent young south Asians from seeking help in community pharmacy settings were discussed, with feelings of shame or embarrassment being most common. Fear of reprisal from friends and family was also stated:

*‘Admitting you have a problem is like embarrassing…almost like you’re letting people down.’*

(Participant D)

*‘Sometimes people might not want to ask for the help that they actually need because they don’t want to be judged by their family or their community.’*

(Participant E)

Concerns about confidentiality were also expressed:

*‘Maybe they’re worried that the pharmacist will go and tell, even though it’s going to be confidential, they might talk to other colleagues to get advice…it may be a bit embarrassing in that aspect.’*

(Participant B)

Most felt that pharmacists could play a role in targeted cardiovascular health promotion via digital media and would be receptive to receiving it:

*‘I think it has to be digital information…social media campaigns I think are better than handing out flyers.’*

(Participant I)

*‘I feel like if there was that education and that awareness surrounding cardiovascular health specifically aimed at that age group, I think that that would be something that I would consider and that probably. Other people would as well.’*

(Participant F)

Upon further questioning, different ways in which community pharmacies could disseminate health advice were also suggested:

*‘Education in secondary schools, universities and like go out and give talks or kind of like local community areas apart from also giving advice in the actual pharmacy’*.

(Participant J)

## 4. Discussion—Key Findings

### 4.1. Culture Clash

Participants in this study reflected experiences reported in earlier studies which found that some young people’s drinking behaviour, particularly young Sikh men, is informed by the actions of friends and family during their formative years [16]. Similarly, most participants verified previously reported ideas that many young people hold beliefs about alcohol and drinking before adolescence [28]. Consumption was reportedly hidden among some young Muslims, who tend to start drinking later than other South Asian groups. This echoed one US-based study which found 60% of Muslim undergraduate students who reported to drink regularly, started after the age of 18 years [29]. Some participants expressed that some South Asian women continue to be expected to abstain from drinking which is in line with previously reported findings [9].

Most notably, excess drinking among Sikh Punjabis was reported. Whilst participants in this study did not provide explicit reasons aside from cultural expectations, previous studies attribute excess consumption among Sikh men to be associated with masculinity [30]. Therefore, variations in drinking behaviours exist among different South Asian communities in the UK, which supports previously reported ideas that any future health promotion must be culturally sensitive [31].

### 4.2. The Great Escape

In the current study, participants cited university as the main place where young South Asians are likely to develop harmful drinking practices. Whilst other UK-based studies confirm that excessive drinking culture is central to the university experience, the main motivating factors previously reported include reducing anxiety, making friends, and general integration [32]. Contrastingly, participants believed that South Asians exhibit such behaviours due to rebellion against their strict upbringing or peer pressure. Participants shared the belief that young people who witness harmful drinking at home are more likely to engage in similar behaviours, reflecting one UK study which found young people who watch their parents drink to excess, more readily engage in similar behaviour [7]. Participants of Punjabi heritage recalled instances where they feared social exclusion if they refused to drink, owing to cultural expectations, which is in line with the literature [16].

### 4.3. Cardiovascular Confusion

Recognition of poor cardiovascular health among British South Asians by young people was good in this study; however, the cardiovascular effects of drinking were poorly understood. Participants readily acknowledged several cardiac co-morbidities that they could link to harmful drinking; however, most were unconfident that they would be able to explain the link to a peer. One explanation which was suggested was inadequate secondary education, which reflects the findings of a recent UK study which found that many young people viewed alcohol-related health promotions from school as insufficient and inapplicable [33]. Therefore, there may be a growing evidence base to suggest that secondary institutions must provide improved alcohol-related health promotion to young people of all ethnic backgrounds, to encourage safe and responsible drinking.

Frequent gym use among young South Asians was emphasised by some participants; however, cardiovascular health was not reported to be the focus and very few participants reported to make any dietary or lifestyle changes to promote better cardiovascular health. Thus, digital health promotion within secondary institutions, about harmful drinking, and cardiovascular health, as part of the NHS’s long-term cardiovascular health plan, should be considered [34].

While participants were largely receptive to pharmacist-led harm reduction, their predominantly healthcare-related academic background may have positively biassed perceptions of pharmacists’ roles, limiting generalisability to non-healthcare students or less health-literate populations.

### 4.4. The Ambiguous Pharmacist

Interviewed participants were receptive to pharmacy-led interventions for alcohol dependence and most would refer a young person seeking harm reduction to a community pharmacist. This reflects one UK-based study of older Caucasian patients who viewed pharmacist-led alcohol dependence interventions positively [35]. Participants in this study identified accessibility as the main benefit of pharmacy-led interventions but also expressed confidence in pharmacists’ skills and training. However, the role of pharmacist in this study was vaguely understood, with most believing signposting to be the only contribution. Currently, harm reduction within community pharmacies consists of indicative screening or opportunistic questioning [19]. This was missed by all participants who had prior healthcare training, indicating there may be a need for greater awareness of these services among the UK public.

Whilst no framework currently supports the delivery of brief interviewing tailored to support young people with alcohol dependence, one recent meta-analysis concluded significant reductions in use can be made by adults up to 12 months post-treatment [24]. Similarly, a recent study investigating patients with a mean age of 19.5 found brief motivational interviewing was effective in decreasing alcohol-related psychological distress [36]. Participants in the current study were mostly receptive to pharmacy-led harm reduction; therefore, pharmacy staff may require future training to address this going forward.

Barriers preventing young South Asians from accessing harm reduction were discussed, including shame, embarrassment, fear of reprisal, confidential and time pressure concerns and lack of confidence with the quality and effectiveness of pharmacist consultations. Similar studies have identified stigma, embarrassment, and fear of punishment as key barriers among adolescents; however, concerns over patient confidentiality are less reported [37]. Efforts must be made to reassure young people that caring and optimistic providers are delivering respectful and confidential services [38].

Findings reflect diverse perspectives within the UK South Asian diaspora, but this may obscure intra-group differences (e.g., Gujarati Muslims vs. Tamil Hindus). Future studies should stratify analysis by sub-group.

### 4.5. Strengths and Limitations

This study provides novel insights into alcohol-related health perceptions among young South Asians in the UK. However, generalisability is limited by the small sample size (*n* = 10), the predominance of female participants (70%), and the overrepresentation of pharmacy students (80%). Participants were also recruited from a single university setting, which may bias findings towards those with higher health literacy and socioeconomic status. These factors likely skew the sample toward a more health-aware and academically engaged group, and the results may not reflect the wider British South Asian youth population. Pharmacy students may be more aware of community health services or more likely to endorse their utility. This limitation was a direct consequence of convenience and purposive sampling within the School of Pharmacy, where the authors had ethical clearance and recruitment access. Although efforts were made to recruit beyond Pharmacy, uptake from other faculties was low. Socioeconomic status was not formally assessed, which limits the interpretability of how economic background may shape drinking behaviours. While we recorded participants’ ethnic and religious self-identification, we did not collect country-of-origin data, which may have introduced heterogeneity in cultural norms related to drinking. Greater diversity in gender, academic background, and educational institutions would strengthen future research.

Additionally, all participants were enrolled in healthcare-related degree programmes (Pharmacy, Dentistry, or Medicine), which may confer higher baseline health literacy and a more favourable view of pharmacists compared to non-healthcare peers. This limits the applicability of findings to the general South Asian youth population. While gender balance was not an inclusion criterion, the predominance of female participants (7 of 10) should be acknowledged. Although no consistent gender-based differences emerged in thematic analysis, the small sample precluded meaningful subgroup comparisons, which may be worth exploring in future work.

### 4.6. Recommendations to Practice and Policy

Increased education about the cardiovascular consequences of excess drinking and general cardiovascular health promotion is needed for South Asians living in the UK. Furthermore, increased awareness of the role of community pharmacies in alcohol-related harm reduction is required, to instil confidence among young South Asians seeking help.

### 4.7. Future Work

Future research should involve a larger, more demographically diverse sample to improve generalisability. A national, multi-site quantitative study using stratified random sampling would enable comparisons across ethnicity, gender, academic background, and socioeconomic status. This would also allow for subgroup analyses (e.g., healthcare vs. non-healthcare students) to explore differences in health literacy, help-seeking behaviours, and attitudes toward pharmacy services. Quantitative methods are justified to measure the prevalence and correlation of key themes identified in this qualitative work, and to assess the extent of knowledge gaps and service preferences across the wider South Asian youth population. Mixed-methods studies may also add value by integrating qualitative insights with generalisable trends. Further studies can include a larger sample size, factoring in equality and diversity, economic and educational status, and, in addition, a non-South Asian comparator group could be considered.

## 5. Conclusions

Young South Asians are aware of the prevalence of poor cardiovascular health within the community, but knowledge of the cardiovascular effects of harmful drinking and the role of the pharmacist in supporting dependence remains inadequate. Thus, increased cardiovascular health promotion, harmful drinking, and awareness of the role of the pharmacist must be developed to potentially reduce unsafe behaviour within this demographic going forward.

## Figures and Tables

**Table 1:** Participant demographics.

Participant ID	Gender	Ethnicity	Religion	Degree Course	Age
A	F	Punjabi	Sikh	Pharmacy	21
B	F	Tamil	Hindu	Pharmacy	21
C	M	Punjabi	Sikh	Pharmacy	22
D	F	Kashmiri	Muslim	Pharmacy	21
E	F	Punjabi Mixed	Not stated	Pharmacy	22
F	F	Punjabi	Sikh	Pharmacy	21
G	F	Tamil	Hindu	Medicine	21
H	M	Gujrati	Muslim	Dentistry	21
I	M	Malayalee	Christian	Pharmacy	22
J	F	Punjabi	Muslim	Pharmacy	21

## Data Availability

The raw data supporting the conclusions of this article will be made available by the authors on request.

## Appendix A. Interview Guide

Knowledge, attitudes, and behaviours towards alcohol consumption and cardiovascular health among South Asians aged 18–25 living in the UK: A qualitative study.

1. Would you like to introduce yourself and tell me a little about your background?

Prompts:

- What course are you studying?

2. Can you think back to your earliest memory of alcohol—how does this experience make you feel?

Prompts:

- Are these feelings largely positive, negative, both or neither, could you elaborate?

3. What are your thoughts on drinking and its prevalence within the South Asian community?

Prompts:

- Can you think of any socioeconomic, family, environmental or other factors that might contribute?
- Do you think there is significant awareness about drinking; what sources of information might be available/where does this awareness come from?

4. What are your thoughts about harmful drinking practices?

Prompts:

- Can you think of any reasons why people may not want to talk about their drinking?
- Can you think of any places such as school, university, the workplace or in the home where young people aged 18–25 may adopt harmful drinking practices?
- Can you think of any circumstances young South Asians may find themselves in where harmful drinking may be promoted?

5. How might harmful drinking at a young adults age, between the ages of 18–25, manifest itself in later life?

Prompts:

- Can you think of any health complications that you could link to harmful drinking?
- Do you think that the drinking behaviours of someone who displays harmful drinking in their youth, gets better, worse or stays the same as they get older—why?

6. To what extent do you think that poor cardiovascular health affects the South Asian community living in the UK?

Prompts:

- Do you think that there is good awareness around preventing poor cardiovascular health within the community—why?
- Do you think that South Asians aged 18–25 living in the UK make conscious decisions to prevent poor cardiovascular health? Could you provide examples?

7. What do you understand to be relationship between harmful drinking and poor cardiovascular health?

Prompts:

- How confident are you that you would be able to explain the relationship between drinking and poor cardiovascular health to someone else—why?

8. If someone aged 18–25 was struggling with alcohol dependence, where would you signpost them to get help?

Prompts:

- Can you think of any healthcare professionals, community leaders or other services that may be able to help them?
- What made you consider this/these?
- What makes them a good candidate to help people struggling with alcohol dependence?

9. What do you understand to be the role of the pharmacist in supporting young South Asians struggling with alcohol dependence?

Prompts:

- Would you consider signposting someone struggling with alcohol dependence to a pharmacist—why?
- What barriers if any might prevent someone from seeking help from their local pharmacist and could you suggest any way in which these could be overcome?
- What role if any, do you think that community pharmacies could play in promoting better cardiovascular awareness to younger audiences to prevent poor cardiovascular outcomes in later life?
